# Evaluation of the stability of implants placed in low-quality bone following the use of osseodensification technique versus ridge expanders:randomized clinical trial

**DOI:** 10.1186/s12903-025-06918-y

**Published:** 2025-09-17

**Authors:** Mariam Samir Nabih, Omar Ahmed Mahmoud Ashour, Waleed Mohamed Abbas, Ahmed ElBarbary

**Affiliations:** 1 Department of Oral Medicine and Periodontology Faculty of Dentistry, Cairo University, Al Saraya Str. 11, Manial, Cairo, Egypt; 2 Department of Oral Medicine and Periodontology, Faculty of Oral and Dental Medicine, Future University in Egypt, Fifth settlement, New Cairo, Egypt; 3 Department of Oral Medicine and Periodontology, Faculty of Dentistry, Ain Shams University, Cairo, Egypt; 4 Oral Medicine and Periodontology, Faculty of Dentistry, Galala University, Suez, Egypt

**Keywords:** Surgical instruments, Bone expansion, Implant stability, Dental implants, Pain

## Abstract

**Background:**

Low bone quality, as well as narrow ridge width, presents a challenge for implant placement and affects implant stability. To date, the effectiveness of a specific technique to enhance implant stability in low-bone quality, along with ridge expansion, is not well established. This study aimed to evaluate implant stability in low-quality bone using osseodensification technique (OD) versus ridge expanders.

**Methods:**

Twenty-two patients with one missing upper tooth and low-quality bone were recruited. The osteotomy site was prepared via either ridge expanders or the OD technique. The assessments included primary implant stability and secondary stability at 3 and 6 months, as well as bone width at different vertical levels (2, 4, and 8 mm), evaluated preoperatively, baseline (immediately postoperatively), at 3 and 6 months. Additionally, pain outcome (PO) was assessed.

**Results:**

Intergroup comparisons of implant stability between interventions revealed no statistically significant differences at baseline or 3 months (*P* > 0.05), whereas there was a statistically significant difference at 6 months (*P* = 0.0268) in favour of the OD group. The PO revealed no statistically significant differences at baseline or after 1 week (*P* > 0.05). The ridge width between interventions showed statistically significant differences at baseline and after 3 and 6 months (*P* < 0.05) at 2, 4, and 8 mm in favour of the OD group.

**Conclusions:**

Considering the significant preoperative difference in crestal ridge width and the short follow-up period, the OD technique appears to be effective in low-quality bone, improving implant stability and enabling the expansion of narrow ridges with minimal postoperative pain.

**Trial registration:**

This study was registered at clinicaltrials.gov on April 2nd, 2022, with the registration number NCT05330546.

## Background

The density of the inner cancellous bone, the thickness of the crestal cortical bone, and their relative distribution throughout the implant recipient site determine the overall quantity and quality of host bone. Poor bone density and inadequate bone volume are primary risk factors for implant failure, as they are associated with increased bone resorption and compromised healing. Notably, the Implant Stability Quotient has been shown to correlate directly with local bone density [1, 2].

Alveolar bone resorption, particularly rapid during the first 2–3 months following tooth extraction, poses additional challenges for implant placement. During this period, both hard and soft tissues undergo dimensional changes. The overall horizontal width loss can reach up to 50% of the original ridge width, with 56% of this resorption occurring on the buccal aspect and approximately 30% on the lingual aspect [3]. To minimize the risk of postoperative bone resorption, a buccal cortical plate thickness of at least 2 mm is recommended when placing dental implants [4].

To improve bone-to-implant contact and avoid the need for additional surgical interventions such as bone grafting, several techniques have been developed. These include the use of osteotomes, ridge expanders, and the OD technique. Other technologies, such as the magneto-dynamic technology using a magnetic mallet, appear promising in safety and patient comfort. However, according to a recent systematic review, this technique requires further investigation [5]. Zarb and Schmitt suggested, based on clinical experience, that a minimum ridge width of 5mm is necessary for ridge expansion [6, 7]. More recent studies, such as that by Koutouzis et al., have indicated that a residual buccolingual bone width of at least 4mm may be sufficient [8].

Osteotomes offer an alternative to traditional drilling techniques, particularly in the maxilla. They are associated with several advantages, including reduced risk to adjacent anatomical structures, minimal heat generation, and the ability to expand narrow ridges [9, 10]. However, their use is technique-sensitive and may generate excessive mechanical stresses, potentially compromising blood flow and causing microdamage to trabecular bone and collagen structures. Additionally, their application in the posterior maxilla can be limited due to restricted mouth opening. Reported complications include concussions and benign paroxysmal positional vertigo [9, 11].

Motor-driven ridge expanders represent another method for ridge expansion. Unlike osteotomes, these devices are inserted via controlled, motor-driven rotation, which reduces surgical trauma and allows for precise manipulation of the expansion site. The thread design of the expanders enables lateral bone compaction during insertion, making them suitable for Type IV bone and other densities where simultaneous expansion and implant site preparation are needed [7, 12].

In 2013, Huwais [13] introduced the osseodensification (OD) technique, a novel approach to biomechanical bone preparation utilizing specially designed burs known as *Densah burs*. These burs merge the benefits of osteotomes with the efficiency and tactile feedback of conventional drilling. They are engineered to preserve and compact bone rather than remove it, leveraging the viscoelastic properties of bone. When operated in a counterclockwise direction, Densah burs can densify bone and expand the osteotomy, enabling implant placement in bone of any density [13–15].

A systematic review by Gaspar et al. [16] emphasized the need for randomized controlled trials to evaluate the clinical efficacy of the OD technique, specifically regarding implant stability and ridge expansion, compared with other conventional drilling techniques. In light of the current knowledge gap, the present study aimed to assess implant stability as the primary outcome in sites with low bone density, comparing the OD technique to motor-driven bone expanders. Secondary outcomes included radiographic changes in ridge width and PO. The null hypothesis stated that there would be no significant difference between the OD and expander groups in improving implant stability in low-quality bone.

## Methods

### Study design and trial registration

This study was designed as a double-centred, prospective, double-blinded, randomized, parallel, two-arm clinical trial. It was registered at clinicaltrials.gov with the registration number NCT05330546 on April 2nd, 2022. The study protocol was approved by the ethics committee of the Faculty of Dentistry, Cairo University, with ID 12,722. The CONSORT guidelines for clinical trials were followed. Every participant completed a written informed consent form after being fully informed of the study’s goals and specifics. The primary outcome of the current study was implant stability; the secondary outcomes included PO and ridge width.

### Sample size calculations

Based on a previous study by Aboelnaga et al. [17], the mean value of secondary stability for implants drilled by expanders was 69.3 ± 2.58. Using a power of 80% and 5% significance, a sample size of 8 implants in each group was needed. This number increased to a sample size of 11 implants in each group to compensate for losses during follow-up. Sample size calculation was achieved via G Power and Sample Size Calculation Software Version 3.1.9.4 (University of Kiel, Germany).

### Patient selection

The present study included a total of twenty-two patients with one missing upper tooth with low-quality bone. These patients were recruited from September 2022 to January 2024 at the outpatient clinic of the Oral Medicine, Oral Diagnosis and Periodontology Department, Faculty of Dentistry, Cairo University, and Future University in Egypt.

The patients met the following eligibility criteria:

### Inclusion criteria

- Single tooth missing in the maxillary region with D3 (350–850 HU) or D4 (150–350 HU) bone [18].
- A residual buccolingual bone width of at least 4 mm at the implant insertion location [8].
- There were no known medications or recognized bone diseases that impact bone metabolism.
- Patients who were hygienic, motivated, and cooperative.
- Patients having an adequate interocclusal space of 8–10 mm [19].

### Exclusion criteria

- Patients with systemic diseases that contraindicate surgery [20].
- Patients who have any habits that might jeopardize the osseointegration process, such as current smokers [21].
- Patients with parafunctional habits that produce overload on implant, such as bruxism and clenching [18].
- Alcohol or drug abuse [22].
- Pregnant and lactating women.

### Treatment allocation and allocation concealment

Patients were randomly assigned to either the test (OD technique) or the control (expanders) group via computer-generated randomization (www.randomizer.org) with a 1:1 allocation ratio, which was performed by the main supervisor (A.B.). Allocation concealment was done utilizing sequentially numbered opaque sealed envelopes that contained the treatment group to which the participant would be assigned. The envelope was opened by the main supervisor (A.B.) after local anesthesia administration at the surgical site. This study is double-blinded because the participants and the evaluator of the study variables and outcomes were blinded. Due to the nature of the procedures, it was not possible to blind the principal investigator (M.S.N) to the treatment protocol.

### Treatment protocol

A thorough history and medical evaluation were performed before the initiation of treatment. Cone-beam computed tomography (CBCT) was done to ensure accurate measurements in three dimensions. All the patients underwent presurgery screening and initial periodontal therapy.

### Surgical procedures for both groups

Local anaesthetics^2^ (lidocaine 2% containing 1:10000 adrenaline) were administered via buccal and palatal infiltration before any surgical procedure. A crestal incision and a mucoperiosteal envelope flap were elevated, exposing the buccal bone and the crest of the ridge. Implant pilot drills were mounted on a low-speed handpiece, and drilling was performed under copious amounts of normal saline solution. Then the principal investigator (M.S.N) was informed by the main supervisor (A.B.) about the technique for delayed implant placement to be performed by opening the labelled envelope, which contained the patient’s number with the assigned treatment.

**Fig. 1:**
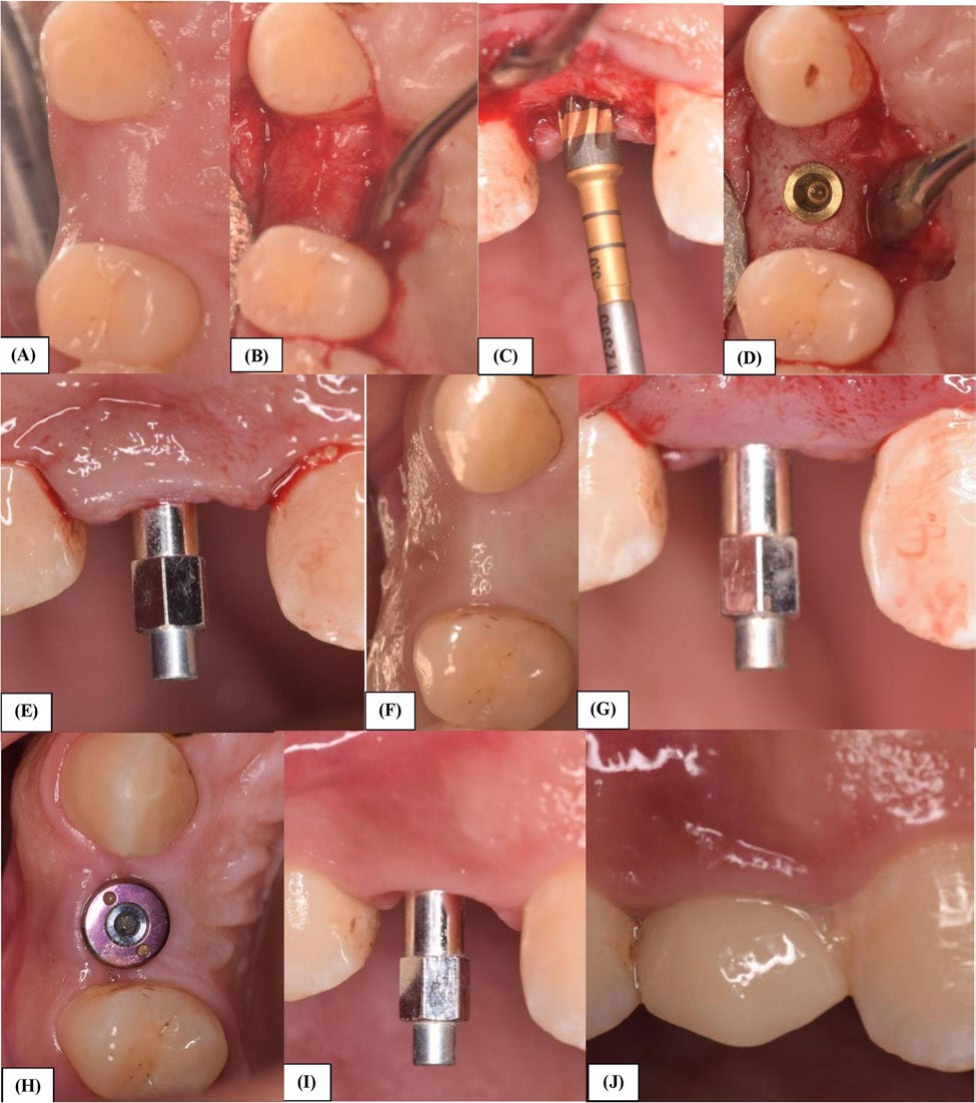
Implant placement in the test group using the Osseodensifying burs. **A** Pre-operative clinical photo of missing 14. **B**: Full-thickness flap reflection. **C**: Densah bur used in drilling for preparation of the osteotomy site. **D**: Implant inserted in the osteotomy site. **E**: Smart peg attached to the implant for measuring the primary stability. **F**: 3-month follow-up. **G**: Smart peg to measure the secondary stability at 3 months. **H**: Healing abutment in place. **I**: Smart peg to measure secondary stability at 6-month follow-up. **J**: Final zircon crown cemented

OD group: The sequential use of Densah burs^3^ according to the ridge width at 800 to 1200 rpm in counterclockwise rotation, to prepare the osteotomy site for the desired implant size (Fig. 1, A-J).

**Fig. 2:**
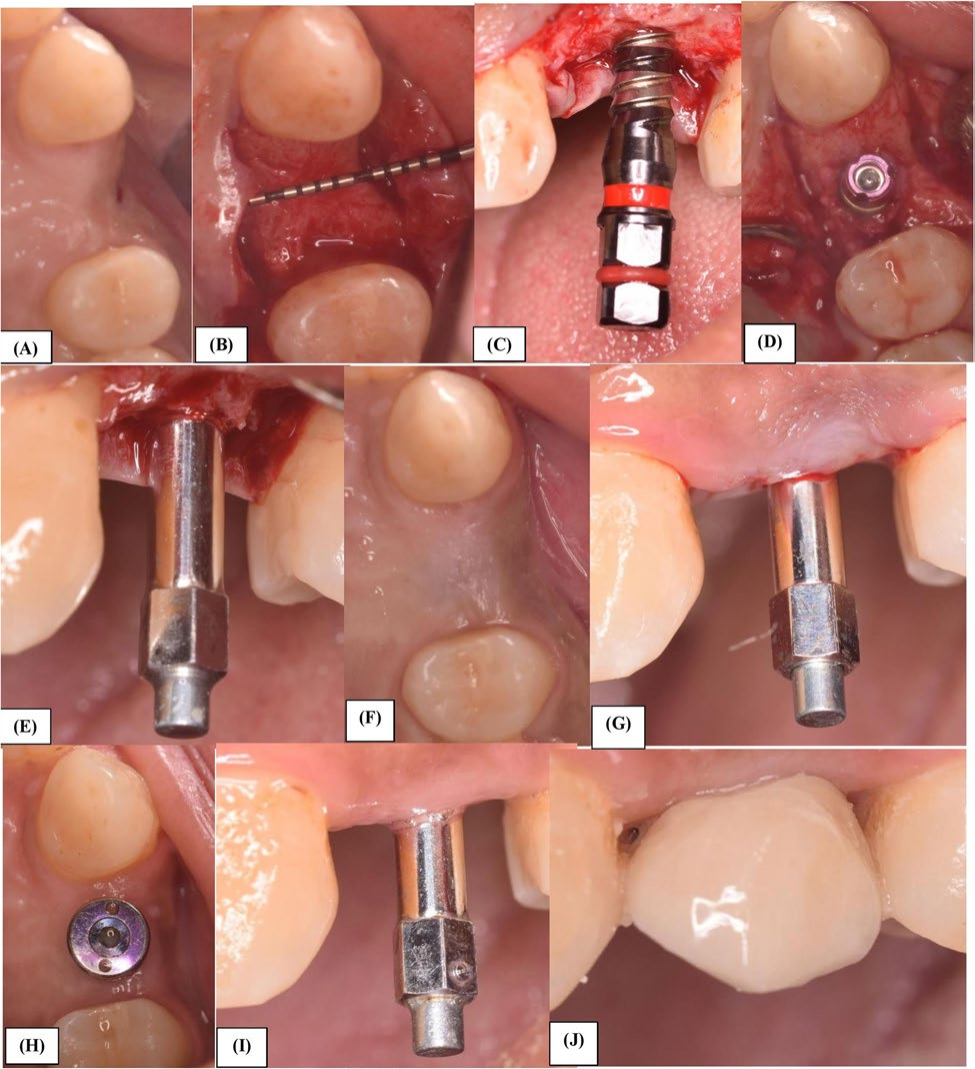
Implant placement in the control group using the expanders. **A **Pre-operative clinical photo of missing 24. **B**: Full-thickness flap reflection.**C**: Expanders used in drilling for preparation of the osteotomy site. **D**: Implant inserted in the osteotomy site. **E**: Smart peg attached to the implant for measuring the primary stability. **F**: 3-month follow-up. **G**: Smart peg to measure the secondary stability at 3 months. **H**: Healing abutment in place. **I**: Smart peg to measure secondary stability at 6-month follow-up. **J**: Final zircon crown cemented

Expanders group: Bone expanders^4^ were used sequentially with increasing diameter (2.6,3.2, 3.6) mm according to the ridge width using an electric handpiece at 50 rpm. Once sufficient resistance was encountered, expansion continued with the aid of a ratchet wrench to prepare the osteotomy site to the desired implant size. The instruments were inserted in intervals, pausing to allow time for the bone to expand (Fig. 2, A-J).

Then, for both groups, the selected implant^5^ size was chosen according to the available bone height and width. The implant was placed in the prepared osteotomy site and turned in a clockwise direction until resistance was encountered. This was followed by using a 2.2 mm hex driver and a ratchet wrench until the implant body was seated within the bone. The assessment of implant stability was performed via Osstell. The cover screw was placed and tightened to seal the internal hex of the implant. The mucoperiosteal flap was repositioned, and suturing was completed via a 4/0 vicryl resorbable suture.

Postoperative care: Antibiotics: amoxicillin (1 g) administered orally two times daily for 5 days. Anti-inflammatory drugs (NSAIDS; ibuprofen whenever needed). Antiseptic mouth rinse: Chlorhexidine mouth rinses were prescribed twice per day for two weeks.

### Clinical measurements

PO was evaluated via a 10 cm visual analogue scale (VAS) assessing pain on the day of surgery and one week postoperatively at the time of suture removal, where patients marked pain severity (0 = no pain, 10 = worst pain).

At the 3-month follow-up, implant stability was assessed via Osstell, the implants were exposed surgically, and healing abutments were placed. One week later, impressions were made, and the final crowns were fabricated and cemented. At the 6-month follow-up, the implant stability was recorded via Osstell.

### Radiographic measurements for ridge width

**Fig. 3:**
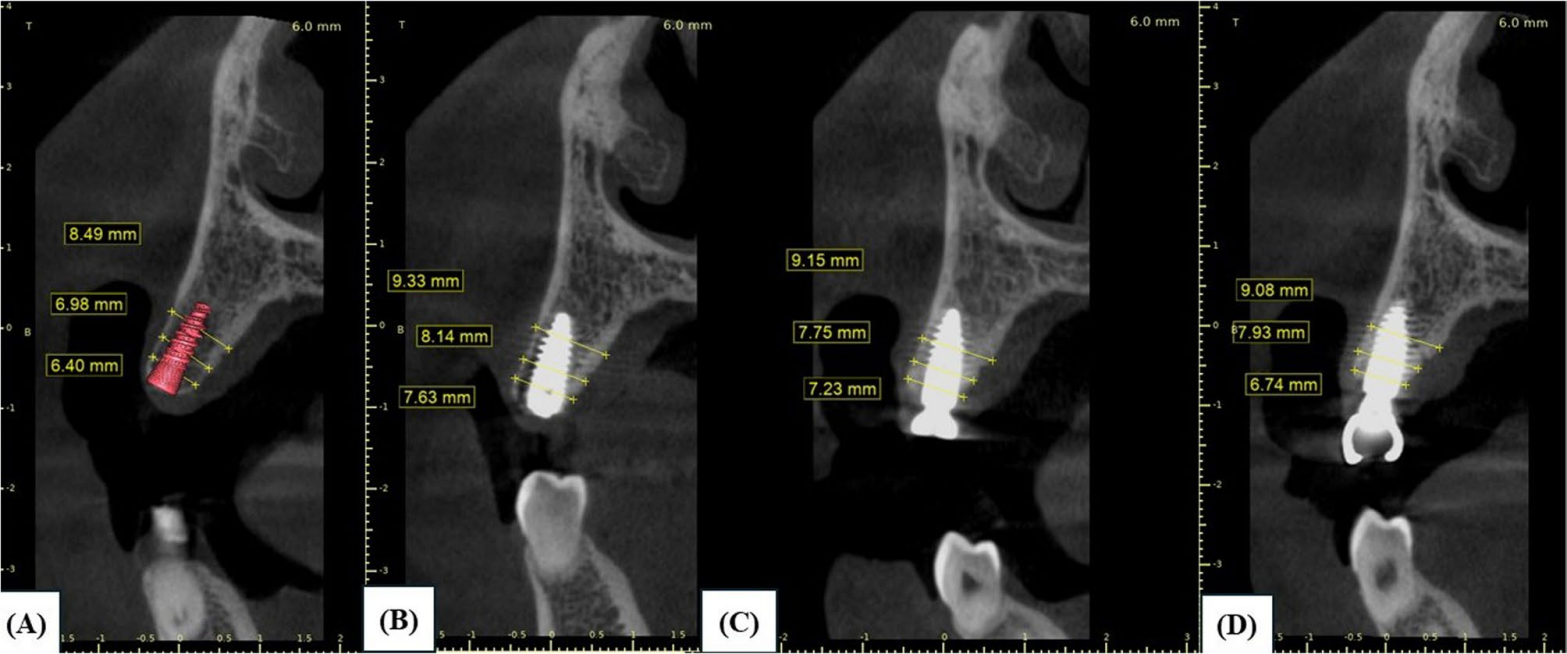
Radiographic assessment of the ridge width at different vertical levels A-D. **A** Before implant placement. **B** Baseline (immediately post-operative).**C** 3-month follow-up. **D** 6-month follow-up

CBCTs were taken before implant placement for implant planning, immediately postoperative, at 3-month, and 6-month follow-up. CBCT scans exposure parameters were as follows: kilovoltage: 90 kVp, milliampere: 12 mA, field of view: 5 × 8 cm, voxel size: 1.5 mm, exposure time: 15.02s. The DICOM files were imported into (Blue Sky plan 4) software, case by case. To ensure reproducibility of the measurements, the sagittal and coronal three-dimensional positions and panoramic curves were adjusted to pass through the centre of the ridge, and the slice thickness was adjusted to 1 mm in all CBCT scans. Accurate comparison between pre- and postoperative data sets was achieved by selecting standardized reference points based on nearby anatomical landmarks at the base of the alveolar process. In the implant planning phase, the implant was placed virtually at the level of the bone crest in the centre of the ridge, setting the implant platform as a fixed reference point throughout all the follow-up periods to measure the ridge width. At the time of surgery, implants were placed at the level of the bone crest as already planned. Subsequently, ridge width was measured at 2, 4, and 8 mm from the implant platform in all time periods (Fig. 3).

### Statistical analysis

MedCalc software, version 22 for Windows (MedCalc Software Ltd., Ostend, Belgium), was used to analyse the data. The Shapiro‒Wilk and Kolmogrov‒Smirnov tests were used to examine the data for normality. The means and standard deviations were used to characterise continuous data that were normally distributed. Independent t-tests were used to compare groups. ANOVA with repeated measurements, followed by the Tukey post hoc test and Bonferroni correction, was used to compare groups within follow-up periods. All tests were two-tailed, and a P value of less than or equal to 0.05 was considered statistically significant.

## Results

### Participants’ flow and demographic results

The present study was conducted on 22 participants whose teeth were missing in areas with low bone quality. After 6 months, 3 implants failed, with an 87% retention rate. Two patients were in the expander group, and one patient was in the OD group. The mean age of the participants in the current trial was 35.00 ± 8.7 years. Baseline characteristics are shown in Table (1).

**Table 1:** Baseline characteristics

	OD group	>Expanders group	*P* value
Male n (%)	5 (45.5%)	4 (36.4%)	0.67
Female n (%)	6 (54.5%)	7 (63.6%)	
Age (years) mean (±SD)	37.36 (±10.33)	32.64 (±6.61)	0.22
Implant site			
First premolar (n)	5	6	
Second premolar (n)	3	1	
First molar (n)	3	4	
Implant length (mm)			
10 (n)	9	8	
12 (n)	2	3	
Implant diameter (mm)			
3.8 (n)	7	6	
4.2 (n)	4	5	

### Clinical results

#### Implant stability

Intergroup comparisons between interventions revealed no statistically significant difference at baseline or 3 months (*P* > 0.05), whereas there was a statistically significant difference at 6 months (*P* = 0.0268) in favour of the OD group. Intragroup comparisons within the OD group revealed no statistically significant change in implant stability with time (*P* = 0.053), and intragroup comparisons within the expander group revealed no statistically significant change in implant stability with time (*P* = 0.166) (Table 2).

**Table 2:** Means and standard deviations of implant stability at different follow-up periods

Stability	OD group		Expanders group		Difference	95% CI	*P* value
	Mean	SD	Mean	SD			
Baseline	75.27	4.15	78.64	7.88	3.36	−2.23 to 8.96	0.2247
3 months	73.10	7.94	73.00	7.73	−0.10	−7.26 to 7.06	0.9770
6 months	79.60	6.36	72.10	7.50	−7.50	−14.03 to −0.96	0.0268*
P value	0.053		0.166				

Means that do not share a letter vertically are significantly different

* Corresponds to a statistically significant difference

#### PO

Intergroup comparisons between interventions revealed no statistically significant differences at baseline or after 1 week (*P* > 0.05). Intragroup comparisons within the OD group revealed a statistically significant decrease in pain with time (*P* < 0.0001), and intragroup comparisons within the expander group revealed a statistically significant decrease in pain with time (*P* < 0.0001) (Table 3).

**Table 3:** Means and standard deviations of pain at baseline and after 1 week

Pain	OD group		Expanders group		Difference	95% CI	*P* value
	Mean	SD	Mean	SD			
Baseline	3.73	1.35	4.82	1.99	1.09	−0.42 to 2.60	0.1480
1 week	0.00	0.00	0.00	0.00	0.00	0.00 to 0.00	1.0000
*P* value	< 0.0001*		< 0.0001*				

* Corresponds to a statistically significant difference

### Radiographic results

#### Ridge width

- 2 mm

Intergroup comparisons between interventions revealed statistically significant differences preoperatively, at baseline, and after 3 and 6 months (*P* < 0.05). Intragroup comparisons within the OD group revealed statistically significant changes in ridge width with time where baseline, 3 and 6 months were statistically significant compared with the preoperative ridge width (*P* < 0.001), and intragroup comparisons within the expander group revealed statistically significant changes in ridge width with time where only the baseline mean was statistically significant compared with the preoperative ridge width, while the 3 and 6 month were non statistically significant compared with the preoperative ridge width (*P* < 0.001) (Table 4).

**Table 4:** Mean and standard deviation of ridge width (2 mm) at different follow-up periods

Ridge width (2 mm)	OD group		Expanders group		Difference	95% CI	*P* value
	Mean	SD	Mean	SD			
Preoperative	6.63^b^	0.93	5.60^b^	0.96	−1.03	−1.87 to −0.18	0.0193*
Baseline	7.74^a^	0.91	6.76^a^	0.92	−0.97	−1.78 to −0.16	0.0213*
3 months	7.64^a^	0.85	5.78^b^	0.98	−1.86	−2.71 to −0.99	0.0003*
6 months	7.73^a^	1.18	5.47^b^	0.87	−2.25	−3.26 to −1.24	0.0002*
P value	<0.001*		<0.001*				

Means that do not share a letter vertically are significantly different

- 4 mm:

Intergroup comparisons between interventions revealed statistically significant differences at baseline and after 3 and 6 months (*P* < 0.05), whereas preoperatively, there was no difference (*P* = 0.2346). Intragroup comparisons within the OD group revealed a statistically significant change in ridge width with time where baseline, 3 and 6 months were statistically significant compared with the preoperative ridge width (*P* < 0.001), and intragroup comparisons within the expander group revealed a statistically significant change in ridge width with time where only the baseline mean was statistically significant compared with the preoperative ridge width, while the 3 and 6 month were non statistically significant compared with the preoperative ridge width (*P* = 0.005) (Table 5).

**Table 5:** Mean and standard deviation of ridge width (4 mm) at different follow-up periods

Ridge width (4 mm)	OD group		Expanders group		Difference	95% CI	*P* value
	Mean	SD	Mean	SD			
Preoperative	7.40^b^	0.95	6.89^b^	1.01	−0.51	−1.38 to 0.35	0.2346
Baseline	8.83^a^	1.12	7.56^a^	0.76	−1.27	−2.12 to −0.41	0.0055*
3 months	8.72^a^	1.15	6.73^b^	0.64	−1.99	−2.86 to −1.10	0.0002*
6 months	8.41^a^	1.00	6.65^b^	0.68	−1.76	−2.59 to −0.92	0.0004*
	<0.001*		0.005*				

Means that do not share a letter vertically are significantly different

- 8 mm:

Intergroup comparisons between interventions revealed statistically significant differences at baseline and after 3 and 6 months (*P* < 0.05), whereas preoperatively, there was no difference (*P* = 0.2346). Intragroup comparisons within the OD group revealed statistically significant changes in ridge width with time where baseline, and 3 months were statistically significant compared with the preoperative ridge width (*P* < 0.001). Intragroup comparisons within the expander group revealed statistically significant changes in ridge width with time, where 3- and 6-months values are statistically significant to baseline values (*P* < 0.001) (Table 6).

**Table 6:** Mean and standard deviation of ridge width (8 mm) at different follow-up periods

Ridge width (8 mm)	OD group		expanders group		Difference	95% CI	*P* value
	Mean	SD	Mean	SD			
Preoperative	7.88^b^	1.34	7.34^ab^	0.97	−0.54	−1.57 to 0.50	0.2942
Baseline	9.35^a^	1.44	7.93^a^	0.64	−1.42	−2.41 to −0.43	0.0072*
3 months	9.26^a^	1.43	7.50^b^	0.74	−1.76	−2.82 to −0.68	0.0029*
6 months	8.85^ab^	1.53	7.19^b^	0.77	−1.66	−2.85 to −0.45	0.0096*
P value	<0.001*		<0.001*				

Means that do not share a letter vertically are significantly different

## Discussion

This randomized clinical trial aimed to evaluate implant stability, PO, and ridge width at various time points following the use of the OD technique versus ridge expanders. The results demonstrated a statistically significant difference in secondary implant stability at 6 months in favour of the OD group (79.60 ± 6.36) compared to the expander group (72.10 ± 7.50). These findings are consistent with those of Aboelnaga et al. [17], who also reported superior implant stability at 6 months using OD (86.40 ± 3.50) versus the expansion technique (69.30 ± 2.58).

In the OD group, implant stability remained unchanged at baseline and 3 months but significantly increased at 6 months. This improvement can be attributed to the biomechanical benefits of the OD technique, which applies gradually increasing forces to compact bone particles into trabecular spaces rather than removing them. In contrast, ridge expanders function by widening the bone without compaction, which may result in plastic deformation and microcracks. These changes can trigger extensive bone remodeling, potentially delaying the transition from primary to secondary stability and leading to reduced implant stability at 6 months [23].

In the present trial, pain levels did not differ significantly between the OD and ridge expander groups immediately postoperatively or at 1 week. These results are consistent with those reported by Rahal et al. [24], who found minimal postoperative discomfort in patients treated with expanders, attributing this to the reduced magnitude of force applied. Similarly, Abdelhamid et al. [25], observed comparable healing outcomes between patients undergoing ridge expansion with motorised expanders and those treated with conventional osteotomes, suggesting that controlled bone compression produces limited soft-tissue irritation. Likewise, Gaspar et al. [26], reported that patients treated with osseodensification experienced minimal pain, postoperative oedema, and analgesic intake. Similar pain profiles and the uneventful healing in our study may be explained by the flap design and the absence of a secondary surgical site, all of which have been shown to reduce postoperative discomfort regardless of the expansion method used.

In terms of ridge width at 2 mm, the OD group showed superior expansion immediately postoperatively compared to the expander group. This contrasts with Tofan et al. [15], who found no significant differences between OD and expanders. They reported mean preoperative ridge widths of 4.20 ± 0.71 mm and 4.52 ± 0.53 mm in the OD and expander groups, respectively, and postoperative widths of 5.48 ± 0.57 mm and 5.71 ± 0.53 mm, respectively. These discrepancies may be due to small sample sizes and variation in group allocation.

At the 6-month follow-up, the OD group maintained a significantly greater ridge width at 2 mm compared to the expander group. In contrast, Tushar et al. [12] found no significant difference, possibly due to limited sample size. Notably, this study is the first to assess ridge width at multiple vertical levels (2 mm, 4 mm, and 8 mm) across different time intervals. The OD group exhibited consistent ridge expansion at all levels, maintained through 6 months, likely due to the design of the OD burs, which evenly distribute stress along the osteotomy walls. Conversely, the expander group showed an initial gain of ~ 1 mm that was not sustained, likely due to thread designs that create excessive compression and stress, especially problematic in D4 bone. This was evident in our study, where two D4 cases were excluded following buccal cortex fractures during expander use [20].

Statistical analysis rejected the null hypothesis, confirming a significant difference between OD and expander techniques in enhancing implant stability in low-density bone after 6 months. However, the results must be interpreted considering the study’s limitations. First, the groups demonstrated a significant difference in preoperative ridge width at 2 mm. In ridge expansion procedures, initial bone width has been shown to influence both the mechanical feasibility of expansion and the biological response. Narrower ridges may require greater force application or incremental osteotomies, potentially increasing cortical microfractures and transient postoperative discomfort [14]. In contrast, slightly wider ridges may allow smoother expansion with less mechanical stress and reduced heat generation, which can promote faster bone healing and better maintenance of primary stability [8]. This imbalance could have acted as a confounding factor, highlighting the need for future studies to control for initial ridge width through stratification or statistical adjustment. Second, the follow-up period was relatively short; longer-term studies are needed to assess implant stability and ridge width maintenance. Third, pain was the only outcome assessed. Future studies should also consider oedema, functional outcomes, and overall patient satisfaction.

Implant failures were recorded in both groups. Two failures occurred in the control group at 3 months due to evident implant mobility. One failure occurred in the test group after implant loading but before the 6-month follow-up. These failures may be attributed to factors such as excessively high insertion torque—common in both OD and expander techniques—which can impair microcirculation and lead to bone necrosis [27], or systemic factors like vitamin D deficiency, which impacts bone quality and osseointegration [28].

In conclusion, the OD technique effectively enhances implant stability in low-density bone for predictable ridge expansion. The results remain stable and consistent over the follow-up periods. Conversely, ridge expanders might be more suitable for cases with moderate bone density (D2–D3) and adequate initial ridge width. Further studies with larger sample sizes are necessary to assess PO.

## Data Availability

The datasets used in the present study are available from the corresponding author upon reasonable request.

## Abbreviations

OD: Osseodensification
PO: Pain outcome
VAS: Visual analogue scale
CBCT: Cone-beam computed tomography

## Footnotes

2 ArtPharma Egypt Pharmaceuticals, 6 th Industrial Zone, 6th of October City- Giza - Egypt.

3 Versah, LLC 2000 Spring Arbor Rd. Suite D Jackson, Michigan 49,203 USA.

4 HaeNaem expander kit 194, Jisan-ro 175beon-gil, Jinwi-myeon, Pyeongtaek-si, Gyeonggi-do, Korea.

5 Roott dental implants **TRATE AG** Bahnhofstrasse 16 6037 Root Switzerland.
